# Adaptation and cross-cultural validation of the United States Primary Care Assessment Tool (expanded version) for use in South Africa

**DOI:** 10.4102/phcfm.v7i1.783

**Published:** 2015-06-19

**Authors:** Graham Bresick, Abdul-Rauf Sayed, Cynthia le Grange, Susheela Bhagwan, Nayna Manga

**Affiliations:** 1 Faculty of Health Sciences, University of Cape Town, South Africa

## Abstract

**Background:**

Measuring primary care is important for health sector reform. The Primary Care Assessment Tool (PCAT) measures performance of elements essential for cost-effective care. Following minor adaptations prior to use in Cape Town in 2011, a few findings indicated a need to improve the content and cross-cultural validity for wider use in South Africa (SA).

**Aim:**

This study aimed to validate the United States of America-developed PCAT before being used in a baseline measure of primary care performance prior to major reform.

**Setting:**

Public sector primary care clinics, users, practitioners and managers in urban and rural districts in the Western Cape Province.

**Methods:**

Face value evaluation of item phrasing and a combination of Delphi and Nominal Group Technique (NGT) methods with an expert panel and user focus group were used to obtain consensus on content relevant to SA. Original and new domains and items with > = 70% agreement were included in the South African version – ZA PCAT.

**Results:**

All original PCAT domains achieved consensus on inclusion. One new domain, the primary healthcare (PHC) team, was added. Three of 95 original items achieved < 70% agreement, that is consensus to exclude as not relevant to SA; 19 new items were added. A few items needed minor rephrasing with local healthcare jargon. The demographic section was adapted to local socio-economic conditions. The adult PCAT was translated into isiXhosa and Afrikaans.

**Conclusion:**

The PCAT is a valid measure of primary care performance in SA. The PHC team domain is an important addition, given its emphasis in PHC re-engineering. A combination of Delphi and NGT methods succeeded in obtaining consensus on a multi-domain, multi-item instrument in a resource- constrained environment.

## Abstrait

**Adaptation et validation de l’Outil d’Evaluation des Soins primaires des Etats-Unis (PCAT version élargie) en vue de son utilisation en Afrique du Sud.**

**Contexte:**

Il est important de mesurer les soins primaires pour faire des réformes dans le secteur de la santé. Le PCAT mesure la performance sur des éléments essentiels pour fournir des soins rentables. Après avoir fait de petits changements avant de l’utiliser au Cap en 2011, on a constaté la nécessité d’améliorer le contenu et la validité cross-culturelle pour une utilisation plus large en Afrique du Sud.

**Objectif:**

Cette étude avait pour but de valider le PCAT développé aux Etats-Unis avant de l’utiliser comme mesure de référence de la performance des soins primaires avant de faire des réformes majeures.

**Cadre:**

Les cliniques de soins primaires du secteur public, les utilisateurs, les praticiens et les gestionnaires dans les districts urbains et ruraux de la Province du Western Cape.

**Méthode:**

Evaluation de la valeur nominale de la formulation de l’article et une combinaison des méthodes du Groupe technique nominal et de Delphi avec un panel d’experts et d’un groupe d’utilisateurs pour obtenir un consensus sur le contenu applicable à l’Afrique du Sud. Des domaines et articles nouveaux et originaux avec un consensus de > = 70% ont été inclus dans le ZA PCAT.

**Résultats:**

Il a été décidé à l’unanimité d’inclure tous les domaines originaux du PCAT. On a ajouté un nouveau domaine, l’équipe de PHC. Trois des 95 articles originaux ont réuni un consensus de < 70%, c.-à-d. de les exclure car ils ne sont pas applicables à l’Afrique du Sud; 19 nouveaux articles ont été ajoutés. Quelques articles ont dû être légèrement reformulés et remplacés par le jargon local des soins de santé. La section démographique a été adaptée aux conditions socioéconomiques locales. Le PCAT pour adultes a été traduit en isiXhosa et Afrikaans.

**Conclusion:**

Le PCAT est une mesure valide de la performance des soins primaires en Afrique du Sud. Le domaine de l’équipe de PHC est une addition importante étant donné son importance dans la reconfiguration du PHC. Une combinaison des méthodes Delphi et NGT a réussi à atteindre un consensus sur un instrument multi-domaine et multipoint dans un environnement aux ressources limitées.

## Introduction

### Measuring primary care performance

Primary health care (PHC), considered the backbone of a country's health system,^1^ is a complex, multifaceted range of activities, a unique integration of knowledge, values and skills drawn from clinical, public health, behavioural and anthropological sciences. In addition to diagnosis, treatment and rehabilitation, the range of skills and activities include person-centred communication, prevention and health promotion applied in a comprehensive family- and community-orientated approach to care. A primary care measurement strategy therefore needs a range of dimensions for it to be a valid measure. Primary care dimensions (e.g. comprehensive care) are themselves multifaceted. Each dimension requires a variety of indicators (items) to describe and measure it (content validity); and item phrasing has to be congruent with the indicator and dimension being measured (face validity).

The Primary Care Assessment Tool (PCAT, http://www.jhsph.edu/pcpc/pca_tools.html) is a multi-dimension, multi-item instrument developed and tested for reliability and validity by Starfield and Shi, Primary Care Policy Center, Johns Hopkins School of Public Health.^2^ The PCAT measures primary care organisation and performance on four core dimensions (access, continuity, coordination, comprehensiveness)and three derivative dimensions (community orientation, family-centredness, cultural competence)known as domains defined in the PCAT manual (http://www.jhsph.edu/pcpc/pca_tools.html). They have been shown to be essential for cost-effective primary care in developing and developed contexts.^3,4,5^

When these essential features are available to primary care users and implemented in their care, the outcomes include improved health and satisfaction, reduced cost, and reduced inequity.^5,6^ The domains are also in line with the Alma Ata Declaration,^7^ universally accepted definitions of primary care,^4,8,9^ and the principles of family medicine,^10,11^ including principles relevant to sub-Saharan Africa.^12^ By surveying the three main primary care stakeholders (users, providers/practitioners and clinic managers) the PCAT measures the extent to which users’ experience of primary care approximates what is essential for cost-effective care.

The extent of primary care clinic adherence in each domain is determined by complex summing of participant responses to a range of items (questions) pertinent to that domain. The PCAT is also able to determine the size of differences between the three stakeholders’ domain scores when the respective user, provider and manager instruments are used. Whilst the dimensions are considered universal, their generalisability may be limited in different cultural and socio-economic settings. Given the focus on health systems strengthening worldwide, it is not surprising that the PCAT is increasingly being subjected to cross-cultural validation to extend its generalisability to countries wanting to align with cost-effective care.^13,14,15,16,17^ There is also evidence supporting the PCAT's ability to measure the impact of changes to PHC systems.^17,18,19,20^

This article describes the process used to adapt and validate the adult expanded version (PCAT AE) for use in South Africa (SA). Whilst there are other measures of PHC that have been found to be valid in contexts other than that in which they originate,^21^ the literature suggests that the PCAT is the most widely used and adapted. Other PHC measures include the Primary Care Assessment Survey (PCAS), the Components of Primary Care Index, the EUROPEP Interpersonal Processes of Care, the Primary Care Evaluation tool (PCET) used mainly in Europe, and the General Practice Assessment Survey (GPAS), all comprising multi-item dimensions.

### Study background

In 2011, following an earlier visit by PCAT author, the late Professor Barbara Starfield, a study team in the Division of Family Medicine at the University of Cape Town conducted a pilot audit of primary care in one Cape Town health substructure (two sub-districts) in collaboration with the service provider, Cape Town Metro District Health Services (MDHS). The adult expanded (AE) version of the original United States of America (USA) PCAT was scrutinised by the study team under the supervision of the author to determine whether changes were necessary prior to local use. Adhering to the author's guidelines for changes to the PCAT domains (B-K)^b^ only minor adaptations were made.^1^ These involved rephrasing some items to improve local comprehension using colloquial phrasing and local healthcare jargon.

Given the high prevalence of tuberculosis (TB) in SA and in the Western Cape in particular, the item screening for lead exposure was replaced with TB screening to improve the content validity of G domain (comprehensiveness − services available). Changes were made to the introduction (A), health insurance (L) and demographic (N) sections to fit the South African context. The adapted version was piloted on 10 patients before the English version was finalised and translated into isiXhosa and Afrikaans to cover the three major languages spoken in Cape Town. Translation included bilingual translators, back translation and piloting with Xhosa- and Afrikaans-speaking users. The translations were further scrutinised by bilingual fieldworkers during their two-day training to administer the PCAT AE. Minor changes were made to the wording of a few items and they were piloted on patients by the fieldworkers as part of their training before the translations were finalised.

The ZA PCAT (AE) 2011was registered with the Johns Hopkins’ Policy Center before starting data collection. All eight clinics in the substructure were sampled. Data from 461 user (patient) PCAT AE questionnaires were entered and analysed. Coding of responses and the analysis was conducted according to the PCAT manual. PCAT Likert scale responses to domain items are coded on a scale of 1 to 4 and 9, with 1 indicating ‘Definitely not’, 2 indicating ‘Probably not’, 3 indicating ‘Probably’, 4 indicating ‘Definitely’, and 9 indicating ‘Not sure/do not remember’. In the analysis 9 is coded as 2 except for comprehensiveness (services provided), where 9 is coded as 0. The score for each domain is calculated by summing all item responses in that domain (with reverse coding where required by the manual) divided by the number of domain items to produce a mean score.

Table 1 shows the results (mean, standard deviation (SD) and range) by domain for the 461 users’ data. Users scored community orientation lowest (mean 2.4) and cultural competence highest (mean 3.5).

**TABLE 1:** Descriptive results of user (patient) scores by subdomain (2011).

Subdomains	Number of items	Mean	s.d.	Range
First contact-access	12	2.5	0.6	1.0–3.8
Ongoing care	15	3.0	0.5	1.3–3.9
Coordination	9	3.4	0.7	1.0–4.0
Coordination (information systems)	3	3.0	0.6	1.0–4.0
Comprehensiveness (services available)	25	3.2	0.5	1.1–4.0
Comprehensiveness (services provided)	11	2.7	0.8	0.0–4.0*
Family-centredness	3	3.2	0.9	1.0–4.0
Community orientation	6	2.4	0.8	1.0–4.0
Culturally competent	3	3.5	0.8	1.0–4.0

s.d., standard deviation.

*, In all subdomains the response code 9 (i.e. not sure/do not remember) was recoded as 2, except for comprehensiveness (services provided) where it was coded as 0 in keeping with the PCAT manual.

The distribution of the dimension (domain) scores confirmed what might be expected locally, providing evidence for the PCAT's construct validity in the South African context. This was further supported by service managers and providers generally accepting the results as a reflection of primary care performance in the substructure. The 2013 study team nevertheless wished to strengthen the content and cross-cultural validity of the PCAT before wider use in SA, and to improve its alignment with provincial and national health plans. The 2011 findings suggested that a few domain items may not have been well understood by primary care users. Although none of the items approximated > 50% ‘Do not know’ responses (requiring adjusted coding and analysis), some were much higher than others. In addition, the users’ PCAT score for ongoing care (domain D) was considerably higher than the provider and manager scores and higher than expected by study team members who had years of experience working in the clinics studied.

A European Union grant was obtained to strengthen the PCAT's validity for South African use and to extend the 2011 pilot measure of primary care performance to other health districts in the Western Cape Province prior to major health sector reform. Study objectives to strengthen the relevance of the PCAT content for SA included improving its face, content and cross-cultural validity by (1) reviewing the phrasing of domain items for user comprehension; (2) reviewing the domains and their content for alignment with the healthcare setting in SA, the Western Cape demographic and socio-economic context, local and national healthcare policies; and (3) improving the 2011 translations.

This study is part of a bigger European Union-funded study in the School of Public Health and Family Medicine at the University of Cape Town which is aimed at improving users’ experience of primary care.

## Research design and method

Approval for the study was obtained from the Human Ethics Research Committee in the University of Cape Town's Faculty of Health Sciences and the Western Cape Provincial Department of Health Research Committee. There were no risks to participants in the study. All data provided were analysed and reported anonymously.

The study was conducted in two parts: (1) an expert panel and a primary care user focus group reviewed the wording of PCAT domain items for their face value; and (2) a combination of modified Delphi and nominal group technique (NGT) methods was used to determine consensus on domain and item relevance for SA. The ZA PCAT was piloted at a primary care clinic before being finalised and translated.

### Part 1: Review of item wording for local use

#### Expert panel (1a)

This panel, consisting of 2011 PCAT study investigators (two family physician researchers, a family physician in charge of clinical governance at a clinic and a fieldworker) had face value evaluation meetings to review the wording of all PCAT items in domains B–K and to rephrase items where necessary to improve comprehension by primary care users in SA. Problematic wording identified in 2011 was given particular attention. Changes were agreed upon by simple consensus. Selected items were referred to the user focus group to assist with improving user comprehension.

#### User focus group (1b)

Two members of the PCAT study team purposively selected and invited six primary care users from amongst patients attending a clinic in the baseline measure study sampling frame to join a focus group. Six consenting adult patients who had attended the clinic at least four times and who were conversant in two of the three main languages spoken in the Western Cape (namely English and Afrikaans) were selected. A modified form of the NGT method was used to obtain group consensus on the phrasing of items selected in part 1a. The process involves each item in turn being presented to the group on a flipchart in one of the two languages. Each participant records her and/or his back translation on a blank sheet without discussion. All responses are recorded anonymously on a flip chart, after which each participant, without discussion, chooses and records the one response that she or he feels most accurately reflects the item presented. Responses (phrasing) achieving > = 70% agreement^22^ are accepted. The steps are repeated for each item. The investigators determine whether the phrasing chosen maintains the intention of the original item; if not, investigators rephrase the item to maintain the original intention, taking into account the information obtained from participants by that stage. The above steps are repeated to determine consensus on the item thus phrased.

### Part 2: Obtaining consensus on domain and item relevance for South Africa

#### Delphi process (2a) (steps 1**–**4 in Appendix 1)

A second expert panel comprising 2 family physicians, 2 clinical nurse practitioners, 2 clinic managers, and 2 family physician educator/researchers were purposively selected by the study team to examine and determine consensus on the relevance of all PCAT domains and domain items (B–K) for the primary care context in SA. The task of each panellist was to determine from her/his own experience, (i) which items were relevant, and (ii) which items, if any, should be added. Background information given to the panellists included a summary of the PCAT, its purpose, and relevance to PHC and family medicine. The panellists were informed of the 2011 PCAT study; that lessons from 2011 were being applied; that rephrasing of some questions might be necessary; and that the process outlined was to validate the ZA PCAT for use in SA by using a combination of the Delphi and NGT methods (Appendix 1). Inviting, consenting and providing information to the panellists were done via email. Panellists were asked to scrutinise all the PCAT domains and their respective items and to score them for relevance on a scale of 0 to 3 (not at all relevant – very relevant). At the end of this stage the emailed data were collated and the percentage agreement calculated for the 9 domains (B–K) and their items. The results together with new items were tabulated.

#### Nominal Group Technique process (2b) (steps 5**–**7 in Appendix 1)

The expert panel in 2a was convened by two study investigators and part 2a results presented (items which achieved < 70% agreement; new items generated; and comments) by displaying them verbatim in poster form for panellists to view and to ensure that their items and comments had been recorded. Three questions were provided to guide the selection and scoring of new items generated in part 2a: (i) Is it important and relevant to primary care?; (ii) In which domain does it belong?; and (iii) Can clinic staff do anything about it? The method requires that the meaning of each new item be clarified and similar items merged (step 5) in a facilitated group discussion. Given that the above discussion may stimulate further thinking on domains and items to include, panellists are given a second opportunity on their own (i.e. NGT silent phase repeated) to generate items they wished to include.

All new items thus generated are in turn clarified to ensure they are understood by all participants and that they differ from existing items (step 5 repeated). These were added to a table along with new items generated in the Delphi process (2a) and original PCAT items that achieved < 70% agreement and scored for relevance as in 2a (i.e. items that achieved < 70% agreement in 2a were re-scored), and were recorded independently. Each panellist then verbally submitted her/his scores in round-robin fashion (step 6). The scores were recorded and summed by the investigators, and the summed scores presented in a projected table (step 7). The percentage agreement for each item was calculated to determine which items achieved consensus (step 7), that is > = 70% agreement. Repetition of steps 3–7 above substituted for the usual successive rounds in the Delphi method, which was limited to one round in part 2a. The percentage agreement for each item is calculated, where 100% = the number of panellists x 3, that is the maximum score per item. Only domains and items on which consensus is achieved (i.e. > =70% agreement) are retained (step 8).

## Results

### Item review and rephrasing (1a and 1b)

Domain and item relevance was not considered at this stage. The phrasing of domain items (questions) considered problematic by the 2011 study teamrequired minimal rephrasing. The team nevertheless wanted user assistance on five questions (D4, F1, G2, J2, K2) to improve user comprehension. These were presented to the six focus group participants for scrutiny as described in 1b of the methods section. Most participants had difficulty understanding the task, especially back translation and its purpose, and preferred to add and discuss questions on problems they encountered with the service. After unsuccessful attempts to explain and guide participants through the planned NGT process, this was abandoned. Instead an open discussion on the phrasing of the questions was conducted to gain as much information from patients as possible for item rephrasing.

### The Delphi process (2a)

Results of the Delphi process (2a) revealed that the expert panel considered all the domains as very relevant; median = 92.6%; range = 48.2% – 100.0%; interquartile range 85.2% – 96.3%. Only 3 of the 95 domain items received < 70% agreement (C6: 48.2 %; C7: 48.2%; H9: 51.9%). No new domains but two new items were added (C4NGT, C5NGT) by the end of this stage.

### The Nominal Group Technique process (2b)

In the NGT process (2b) a total of 19 new items were generated and scored, as described in methods sections 2a and 2b. The results are presented in Table 2. These items related to the first contact – access (C), ongoing care (D), coordination (E), comprehensiveness (services available [G] and services provided [H]), and culturally competent (K) domains. One new item, the PHC team, emerged during clarification of newly generated items. The panel felt that it was important to add the PHC team as a domain instead,given the emphasis on the PHC team in SA'sdistrict health policies and plans. A list of items to describe the composition of the PHC team in a comprehensive primary care service was generated. PHC team items were agreed on by simple consensus and not subjected to the NGT stepped process due to time constraints.

**TABLE 2:** Rescoring of items scored < 100% in part 2a and scoring all new items (parts 2a and 2b).

Item code	Domains and items (questions)	% agreement
C. First contact – access		
C13NGT	Are the signboards (signage/instructions) at your CHC clear?	100
C14NGT	Is the staff friendly and approachable?	100
C15& 16NGT	Is it easy to lay a complaint or compliment or make a suggestion at your CHC? (C15)	
Is there a complaints/suggestion box at your CHC? (C16)	100	
C4NGT	Are you able travel safely to your CHC?	67
C5NGT	Is it difficult to get to your CHC? Yes/No. If yes, please explain.	57
C6NGT	How long does it take you to get to your CHC?	Removed by simple consensus
C17NGT	How much does it cost you to get to your CHC?	86
C6*	When your CHC is closed on Saturday and Sunday and you get sick, would someone from there see you the same day?	46
C7*	When your CHC is closed and you get sick during the night, would someone from there see you that night?	54
C5	When your CHC is closed is there a phone number you can call when you get sick?	71
C11bNGT	Is it difficult for you to get a second opinion when necessary?	81
D. Continuity of care (ongoing care)		
D15b& cNGT	If response to D15 is 4 or 3, then: ‘Where would you go?’ (D15b)	90
	If response to D15 is 4 or 3, then: ‘Why would you change?’ (D15c)	
D4	If you have a question about your health, can you phone your CHC and talk to the doctor or nurse who treated you before?	83
D14	Can you change your CHC if you want to? (Retained because D15b and c were added)	67
E. Co-ordination		
E9bNGT	How long did it take for you to be given your appointment by your CHC?	95
E9cNGT	From the time that you were given your appointment date, how long before you actually saw the specialist?	90
F. Co-ordination (information systems)		
E14NGT	Would the CHC assist you to get medical-legal or insurance reports if required?	71
G. Comprehensiveness (services available)		
G26NGT	Checking for weight problems?	95
G27NGT	Access to termination of pregnancy services at or via your CHC if required?	100
H. Comprehensiveness (services provided)		
H15NGT	Advice and treatment on sexually transmitted infections	100
H9*	Ask if you have a gun, its storage or its security	10
K. Culturally competent		
K4bNGT	Do you think your CHC understands/respects your culture?	100
K4cNGT	Do you feel comfortable discussing religious or cultural issues that affect your health with the staff at the CHC?	100
P. PHC team (new domain) (items agreed on by simple consensus)		
P1.	Can you see a social worker if you need to? E.g. for help with counselling for a family problem or advice about social services?	-
P2.	Can you see a physiotherapist (and occupational therapist) at your CHC if you need to? E.g. to help with muscle sprains or movement following a stroke.	-
P3.	Can you be visited in your home by a community health worker linked to your CHC if you need it? E.g. for home-based care for TB, HIV or basic care such as wound dressings.	-
P4.	Can you be seen by a health promoter/dietician for advice on these topics?	-
P5.	Can you be seen by a mental health worker at your CHC for help with any mental health problems?	-
P6.	Can you be seen by a dental/oral health worker at/or linked to your CHC if you need it? E.g. any problems with your teeth.	-
P7.	Can a child (under 12 yrs) be seen at your CHC?	-

ZA PCAT validation.

DELPHI-NGT comments and additional questions.

7 Participants; score 0–3; max score per item = 21, that is 100% agreement.

NGT, items added via Delphi-NGT process; CHC, community health centre.

*, Retained in the printed version; explained in discussion section.

This was followed by a final round of scoring (individually and without discussion), which included all the original PCAT items which received < 100% agreement in 2a and all new items generated as described above. Whilst rescoring the few original items that achieved < 100% but > 70% in part 2a was not essential, the panel felt that a final round of scoring that included these items should be the final step. Table 2 shows the final percentage agreement for all original items which achieved < 100% agreement, all new items (coded as ‘NGT’) as well as the PHC team domain.

Following piloting of the ZA PCAT AE 2013 on 10 patients at a clinic in the baseline study sampling frame, minor changes were made to the phrasing of a few items. This completed the ZA PCAT validation process. All domains and items achieving consensus at the end of part 2b constitute the ZA PCAT AE 2013.^2^ The rationale for retaining original PCAT items which did not achieve consensus^b^ for use if needed is explained in the discussion below. The provider (PE) and manager (FE) instruments were accordingly aligned with the ZA PCAT AE by the study team. The additions to the English AE version were translated and back translated for the isiXhosa and Afrikaans versions and existing items in the 2011 translations were reviewed by bilingual study team members before being finalised.

## Discussion

Following lessons from the 2011 pilot study, we sought to validate the PCAT content and cross-cultural applicability for use in SA, that is improve its relevance to primary care in SA and its comprehension in three languages before extending the audit to other health districts. Minimal item rephrasing was necessary following that done in 2011. The fact that no domains were removed, only one new domain was added, only three items achieved < 70% agreement, and a high median percentage agreement on domain and item relevance was achieved, indicates the high content validity of the original PCAT for SA.

The silent phases in parts 2a and 2b ensured that participants were able to generate items and to agree/disagree independently of each other. The discussion during the clarification stage and the range of scores in part 2b suggest that participants were not unduly led by each other's views or scores. The addition of the PHC team domain aligns the PCAT with the emphasis on the PHC team in local and international policy documents and research.^23,24,25,26,27^ In a study identifying key principles of family medicine in sub-Saharan Africa, practising as members of a PHC team emerged as important and included nurse practitioners.^12^ Including the PHC team as a domain in the ZA PCAT is therefore likely to be supported by primary care physicians in Africa.

Nurse practitioner (CNP) was not generated as a PHC team item. This can be explained by the fact that PHC in SA is a nurse-led service.^23^ In most primary care clinics CNPs are the only clinical practitioners. Where there are doctors, CNPs practice as members of the clinical team alongside them.

The PHC team domain uses the same Likert scale for rating responses to items and is analysed in the same way as existing domains. PHC team scores are included in the total primary care score – a summing of all the domain scores. New items which do not use the PCAT Likert scale (patient waiting times, travel costs and patient satisfaction) are included under the relevant domains but are analysed separately. The study team elected to retain original items C6, C7, H9 in the printed version^b^ used for the data collection in order to allow for international comparison of results where the original PCAT is used. Analysis of the data will include or exclude these items as required. Cronbach alpha and factor analyses on the ZA PCAT 2013 will be included in the second article reporting the results of the PCAT survey of users, providers and managers.

The method used to improve the wording of domain items for better comprehension by users did not follow the standardised seven-step method described by Sousa and Rojjanasrirat^28^ in their review of methodological approaches to translation, adaptation and cross-cultural validation of research instruments; also used in the Korean adaptation of the PCAT.^15^ A key component of our cross-cultural method was the user focus group (part 1b). However, most of the participants had difficulty understanding the task, especially back translation and its purpose, which led to the planned NGT process being abandoned. Users’ preference for raising and discussing problems they encountered with the service is highlighted in patient satisfaction surveys.

The research team nevertheless constituted an expert panel with years of experience practising and teaching in local primary care services. The same team conducted the 2011 study and included a research assistant with experience in developing and administering questionnaires in other research projects in local primary care services. The combined experience was applied in updating and improving the translations of the English PCAT into the two other main languages spoken in the Western Cape. Edits to the demographic section (N) to align it with Western Cape demographic and socio-economic features were also made. Even though the translation method did not follow all the steps suggested by Sousa and Rojjanasrirat,^28^ the bilingualism advised for cross-cultural validation was well represented on the panel and the research assistantwas trilingual.

The pilot conducted on 10 patients before finalising the ZA PCAT AE 2013 will have reduced the impact of our failure to achieve the primary objective of the focus group, by providing another opportunity to identify difficulties with phrasing and administering the PCAT to users. In addition, the 2011 translation conducted by the study team was also scrutinised by the bilingual fieldworkers. Their practical training on patients served as a second pilot of the isiXhosa and Afrikaans translations in 2011.

The focus group experience nevertheless provided important insights, including the depth of feeling amongst users about their frustrations with primary care; the challenges faced by users who are functionally illiterate (which complicated the task of the researchers in this context); the importance of a participatory method when involving user stakeholders and the need for researchers to be flexible. The communication challenge between informant and researcher reflects a common feature of the primary care consultation, namely the potential conflict between patient and practitioner agendas, where ‘give and take’ is required to achieve a therapeutic partnership.

Combining the Delphi and NGT methods in a two-stage process had the benefit of participants independently generating responses in at least one round before meeting for the NGT, thus reducing the impact of attrition of participants and delayed responses that can bedevil a Delphi process. The combination of the Delphi and NGT methods may be better considered a modification of the NGT.^22^ In verbal feedback at the end of part 2b the expert panel reported that, as busy clinicians and managers, they found the combination of Delphi and NGT methods useful in seeking consensus on the content and wording and preferred to meet once-off rather than have a number of iterations as required by the Delphi method. They also preferred the opportunity to interact with each other, especially in the prioritisation and clarification stages of the NGT.

In addition, the modification provided the opportunity for doctors, nurse practitioners and managers to contribute as equals in a key stakeholder group. Modelling such a method that can be used in clinics by managers and practitioners to obtain multi-stakeholder consensus on complex activities and interventions in a time-constrained and limited-resource context may also be useful. Involving these key stakeholders should also increase the likelihood of the ZA PCAT's use in ongoing monitoring, evaluation and quality improvement, also guiding the revitalisation of PHC and assisting health sector reform in SA. Whilst the study did not specifically address aligning the PCAT with provincial and national health policies and plans, it can be argued that including managers, practitioners and educators with years of experience in health services in SA guided the work with these in mind – evidenced by the addition of the PHC team.

As noted above, the findings concur with key principles of family medicine in sub-Saharan Africa and therefore point to the potential for the ZA PCAT to be similarly used in other parts of Africa, as well as to measure the impact of interventions aimed at strengthening PHC systems. This includes measuring the impact of postgraduate training in family medicine (primary care physicians) on person-centred comprehensive, community-based primary care as promoted in the Victoria Falls Statement.^24^ Given that primary care in SA is nurse-driven, the finding that the PCAT content is highly relevant to SA suggests that CNPs should also be trained to apply the essential elements of primary care. If the benefits of a PHC team are to be realised, training in the family medicine approach to primary care – currently the preserve of primary care physicians – should be extended to CNPs. Primary care physicians and CNPs should be trained together on the content, application and measurement of the essential dimensions of primary care. Primary care facility and district managers should be aware of the importance of these elements and trained in methods that improve access to them when allocating and managing resources. They should also be trained in the use of the PCAT to monitor the organisation and performance of the primary care services they manage.

Limitations of the cross-cultural validation method used, when compared with those suggested by a scholarly review,^28^ are discussed above. This will need to be considered if there are significant differences in scores between the three language groups after the baseline results are analysed. Other study limitations include the expert panel and patient informants being local only. The full Delphi method would have permitted wider representation on the expert panel and could have included participants in other sub-Saharan countries. The items in the PHC team domain do not describe team functioning or the quality of team-based care and therefore limit the potential value of this measure. Items that describe team function should be developed and added in future studies.

Is the ZA PCAT 2013 suitable for the Western Cape only? Given the diversity across the nine provinces in SA, translation into local languages and some rephrasing will be necessary, along with changes to the demographic section, depending on region or province. However, we think it unlikely that major domain and item changes will be necessary.

These limitations notwithstanding, the findings are in keeping with those of PCAT validation studies in other countries^15,16,17,18,19,20^ where the essential features of primary care measured by the PCAT were also found to apply. The Brazilian study^18^ kept closely to the original items, whereas other country studies removed a number of domain items, such as in the Chinese PCAT.^17^ Haggerty et al.^21^ examined the validity of a number of instruments that measure PHC from the user perspective in the Canadian context, including the PCAT, the PCAS, the Components of Primary Care Index, and the EUROPEP Interpersonal Processes of Care – all composed of multi-item dimensions. The study found that these instruments performed similarly in Canada as in their original contexts. The Brazilian PCAT study is of note for SA. It showed that the PCAT also applies in a developing context.^18^ The PCET, not included in the Haggerty study, is also of interest.^29^ It includes the four core primary care dimensions measured by the PCAT (access, continuity, comprehensiveness and coordination) as well as four health system functions (financing, creating resources, stewardship and delivery services) that are worthy of assessment in a primary care audit. These functions can be included alongside a PCAT audit without having to adapt the PCAT.

Other audit instruments used in SA currently include the National Core Standards (NCS) instrument^26^ and the chronic diseases audit tool used in the Western Cape. With respect to possible duplication, whilst the NCS includes items on patient-centred care, they are not used to assess relational continuity (PCAT ongoing care [D)] domain), a multifaceted core primary care dimension. The NCS does not seek to determine performance on the other key dimensions of the primary care process, but focuses instead on infrastructure, equipment and administrative resources required.

Similarly, the chronic diseases audit tool–currently a record audit of selected indicators of common chronic disease care– is not a measure of comprehensive PHC. Regarding patient satisfaction surveys, used as a means to determine patients’ views on their health care, there may be some item overlap. However, the PCAT is not a patient satisfaction questionnaire. It is, rather, an evidence-based measure of performance on features of primary care demonstrated to be essential for cost-effective care. Likewise, patient health survey instruments such as the SF-36^30^ are limited to evaluation of health and not a measure of the range of dimensions necessary for cost-effective primary care.

## Conclusion

This is the first of two articles reporting PCAT studies in Africa. It describes the content and cross-cultural validation of the PCAT for use in SA. The results suggest an important role for the PCAT in the Western Cape Province and nationally, given the South African National Health Policy's imperative of PHC re-engineering and broader health sector reform.^23^ Future audits of primary care performance should include private sector services. The findings have implications for the training of primary care doctors and nurse practitioners as well as clinic and district managers.

Further research should include strengthening the PHC team domain to enable assessment of team functioning and performance.

A second article will describe the baseline results of the ZA PCAT 2013 study in the Cape Town MDHS and the Cape Winelands, a rural district.

## Footnotes

1 Edits to item phrasing were made in B2, B3, C3, C6, C8, C9, D7, D12, E1, E2, F1, F3, G2, G3, G10, G12, G13, G14, G15, G18, G20, G21, G22, G23, G25, H2, H8, H12, J1, J2, J3, J18, K2. Local vernacular was used and / or examples added in order to improve user understanding, e.g. ‘prenatal’ replaced with ‘antenatal’ (G20), and ‘blood or sputum’ added to E1 as examples of laboratory tests. In G9, screening for lead exposure was replaced with TB screening. Changes to the introduction and screening (A), insurance (L), and demographic (N) sections were not subject to the guidelines and were made independently by the team to suit the local and SA context. Cross-cultural improvements to demographic and socio-economic sections included adding items on home language, type of housing, sanitation, level of education and household income.

2 South African Adult Primary Care Assessment Tool (ZA PCAT AE 2013), adapted from the original PCAT by the South African PCAT Study Team, Division of Family Medicine, School of Public Health and Division of Family Medicine, University of Cape Town, 2013. Original Adult Primary Care Assessment Tool – Expanded Version developed by Barbara Starfield, Primary Care Policy Center, Johns Hopkins University 1998 (for access to the document, please contact corresponding author).

## APPENDIX 1: Information given to expert panellists (Phase 2).

Consensus method and outline of the process

You are being presented with the user (patient) version of the PCAT only. The same domains and similar questions (items) are asked of clinic practitioners and managers in the practitioner and manager PCAT versions, but from their respective perspectives. Within each domain, a number of questions (domain items) to users of primary care services determine whether that domain is present (accessible) and applied (utilised) in their care. We want to know which questions may not be relevant for determining quality primary care in SA given your understanding of patients’ and communities’ health needs; your experience managing common presenting problems in their context; and whether any additional questions should be added.

**Part 1 (steps 1–3)** will be conducted on your own at the end of which you email your responses to the study team.

**Part 2 (steps 5–7)** will be conducted with the panellists meeting as a group. We envisage that each part can be done in 90mins or less. By the end of step 7 an enhanced ZA PCAT will have been agreed on via the consensus process outlined.

The attached PCAT questionnaire has 9 primary care domains (essential elements) each with a number of items posed as questions to describe the presence and practice of each domain. You are being asked to:

- rate the relevance of domain items on a scale of 0–3.
- add any domains and / or items that you feel are important in the SA context that are missing in the current version.

(comment on italicised text if you wish and have the time)

The ratings of the expert panel will be combined to obtain an overall score for each item; depending on the scores, domains items will be retained, removed or added. Items that receive consistently low scores will be removed at the end of part 1. The remaining and/or additional items will go into round 2 and follow the same process above. Further rounds will follow if necessary until consensus is achieved by the panel on which items should remain, which should be removed and which if any items or domains should be added. Consensus is defined as 70% agreement and will constitute the revised ZA version of the PCAT to be used in the study.

Expert panel: Combined Delphi and Nominal Group Technique method:

**PART 2a**: Steps 1–4 are conducted independently via email prior to the expert panel convening:

1. Introduction and aim of the exercise
  The purpose and objectives of the consensus method have been explained above.
2. Presentation of the questions and definition
  The domain items (questions) for scoring are as per the attached ZA PCAT Adult Expanded (AE) version along with the definition of each domain. Score the relevance for each domain item in the electronic document.
3. The ‘silent’ phase – scoring the relevance of each domain and items and generating new domains and items
  3.1 Before you score the domain items, please read the definition of that domain and keep it at hand to refer to as needed.
  3.2 Score the relevance of domain items (questions) from your knowledge and experience of what is required for good comprehensive primary care in SA on a scale of 0–3 where 0 is definitely not relevant, i.e. not at all relevant; and 3is definitely relevant, i.e. totally relevant.
  3.3 At the end of each domain, add any item(s) not currently in the ZA PCAT that you feel should be included in that domain. You do not need to number them. Please indicate briefly next to any new items, why you would think these should be added.
  3.4 In addition, after scoring all the domains, add any other domain(s) (in the box at the end of the document) that you feel should be included and items that will determine the presence (access to) and utilisation (application) of that domain. Indicate briefly next to any new domain(s), why you think it should be added. You may add a domain even if you do not have items to describe it.

***Submit the document with your scores and any additional items (i.e. responses to step 3) to the investigators (Graham Bresick<graham.bresick@uct.ac.za> and Nayna Manga <nayna.manga@uct.ac.za>) via email by 19 April 2013***

4. Prioritisation
  All the panellists’ responses will be captured, analysed and prioritised by the study team in preparation for Part 2 below.

**PART 2b:** Expert group convenes

**Introduction**

Participants and investigators are introduced. A brief overview of the purpose, objectives and process of the consensus method is given and any questions for clarification dealt with.

5. New item presentation, clarification and rationalisation
  Any new items and domains generated in step 3 are presented to the expert panel and their meaning clarified in the group to ensure they are understood by all and how they differ from existing items. New domains and items with similar meanings and intentions will be merged. The relevance of items is not discussed at this stage. This step ends with a second list of new domains and items.
6. ‘Silent’ phase rating of new domains and items
  Each participant scores each domain and item in the list generated in step 5 above on his/her own on a sheet of paper as in step 3.2. *(Highlight new items in their respective domains).* The scores are submitted without discussion and analysed (prioritised) by the investigators to determine which from list will be added.

Presentation of final list to the panel

All domains and items scored as relevant and not relevant in step 3.2 in part 1 and step 6 in part 2 are presented. Any ‘borderline’ items are noted and can be re-scored - again individually and without discussion.

All the domains and items thus scored as relevant for inclusion will constitute the final content of the ZA PCAT 2013 i.e. > = 70% of the maximum score = consensus

Compilation of ZA PCAT v2
  The PCAT study team compile ZA PCAT 2013 according to steps 1–7 in parts 1 and 2 above.

Participants are asked to record two things they found useful and two they did not find useful about the process. Part 2 ends with any general discussion the panel may wish to have.
